# The application of machine learning approaches to determine the predictors of anemia among under five children in Ethiopia

**DOI:** 10.1038/s41598-023-50128-x

**Published:** 2023-12-21

**Authors:** Abdulaziz Kebede Kassaw, Ali Yimer, Wondwosen Abey, Tibebu Legesse Molla, Alemu Birara Zemariam

**Affiliations:** 1 Department of Health Informatics, School of Public Health, College of Medicine and Health Sciences, Wollo University, Dessie, Ethiopia; 2 Department of Public Health, College of Health Sciences, Woldia University, Po. Box: 400, Woldia, Ethiopia; 3 Department of Information Technology, College of Informatics, Wollo University, Dessie, Ethiopia; 4 Department of Pediatrics and Child Health Nursing, School of Nursing, College of Medicine and Health Sciences, Woldia University, Woldia, Ethiopia

**Keywords:** Diseases, Health care, Medical research, Risk factors

## Abstract

Health professionals need a strong prediction system to reach appropriate disease diagnosis, particularly for under-five child with health problems like anemia. Diagnosis and treatment delay can potentially lead to devastating disease complications resulting in childhood mortality. However, the application of machine learning techniques using a large data set provides scientifically sounded information to solve such palpable critical health and health-related problems. Therefore, this study aimed to determine the predictors of anemia among under-5 year’s age children in Ethiopia using a machine learning approach. A cross-sectional study design was done using the Ethiopian Demographic and Health Survey 2016 data set. A two-stage stratified cluster sampling technique was employed to select the samples. The data analysis was conducted using Statistical Package for Social Sciences/SPSS version 25 and R-software. Data were derived from Ethiopian Demographic and Health Survey. Boruta algorism was applied to select the features and determine the predictors of anemia among under-5 years-old children in Ethiopia. The machine learning algorism showed that number of children, distance to health facilities, health insurance coverage, youngest child’s stool disposal, residence, mothers’ wealth index, type of cooking fuel, number of family members, mothers’ educational status and receiving rotavirus vaccine were the top ten important predictors for anemia among under-five children. Machine-learning algorithm was applied to determine the predictors of anemia among under- 5 year’s age children in Ethiopia. We have identified the determinant factors by conducting a feature importance analysis with the Boruta algorithm. The most significant predictors were number of children, distance to health facility, health insurance coverage, youngest child’s stool disposal, residence, mothers’ wealth index, and type of cooking fuel. Machine learning model plays a paramount role for policy and intervention strategies related to anemia prevention and control among under-five children.

## Introduction

Machine-learning algorithm, which is a subset of artificial intelligence, plays a significant role to discover new medical knowledge and showing new ideas to practitioners and specialists. Predictions of diseases has a significant role in data mining ^1^. There are two groups of data mining tasks. The general features of an existing data can be described by descriptive data mining. Whereas predictive data mining tasks predominantly attempt to perform predictions according to the inference results of available data using two kinds of machine learning methodologies, named supervised and unsupervised methodologies^2,3^.

Health service institutions contain sensitive and very large data, which require a careful handling mechanisms. Health professionals need a strong and best prediction system in order to reach appropriate disease diagnosis, particularly for under-five child with health problems like anemia. Diagnosis and treatment delay can potentially lead to devastating disease complications, which results in childhood mortality. However; machine-learning approaches play the paramount roles to solve such palpable critical health and health related problems ^2^.

Anemia approximately affected 1.62 billion people globally^4^ and 36.4–61.9% of under five children in sub-Saharan Africa^5^. About 293.1 million (47.4%) under-5-year children were anemic Globally in 2017, and out of these, 67.6% of the children were in Africa^6^.Globally, 9.6 million children are approximately severe anemic^7^. It is the burning problem of both developed and developing countries^8^.Similarly, anemia is a devastating public health problem affecting approximately 83.5 million children in Sub Saharan Africa and its prevalence was 67%^9^.

According to the Ethiopian Demographic and Health Survey (EDHS) report; the prevalence of anemia among children is 57% In Ethiopia^10^.

There are different risk factors for anemia across nation to nation and region to region; such as nutritional deficiencies, intestinal worms, HIV infection, malaria, chronic diseases like sickle cell disease and hematological malignancies^9,11^.

Globally, there are a number of devastating consequences of anemia, mainly related to economic and social development. Morbidity and mortality increment is also its fatal consequences^12^. Low tolerance to infection, decreased mental performance, and anemia-related heart failure morbidity and mortality are the main destructive consequences of anemia among under-five children^13,14^.

Low income families’ under-five age children are more vulnerable for developing anemia as a result of iron deficiency following high iron demand during the rapid growth period, although anemia affects all age group^15^. Anemia is the burning problem in Sub-Saharan African Countries such as Mali 55.8%^16^; Kenya 48.9%^17^ and Tanzania 79.6%^18^. Lack of mothers’ knowledge about the problem^19^, low iron bioavailability of the diet^20^, poor nutritional practices and unhealthy food habits^21^, decreased physical activities^14^ and parasitic infestations are additional factors for hemoglobin (Hgb) level decrement in children^22^. Factors including low socio-economic status, family size, ignorance, and illiteracy are associated with anemia among under-five children. Intestinal helminthic and Hook Worm Infections are the causes of gastrointestinal blood loss, finally ending up with iron store depletion and gradually also leads to erythropoietin impairment^23^. This results in mal-absorption and loss of appetite, finally resulting in micronutrient deficiency worsening and children anemia^13^.

The prevalence of anemia in under-five children significantly varies across nation to nation and region to region in particular. These differences can be due to the mother, the child, environmental variables, nutritional deficiencies, intestinal worms, HIV infection, malaria, and chronic diseases like sickle cell disease. The socioeconomic and demographic profiles which are associated with anemia among under-five age children in Ethiopia have been extensively studied in the past using conventional regression models. The prevalence of anemia and its determinant factors among under-five age children has been examined by several studies using common cross-sectional statistical methodologies in various regions of Ethiopia. Majority of the previous researches used small-scale data and less number of risk factors, which were restricted to a single district or city.

Effective decision-making is the ultimate result of reliable information through careful and appropriate examination and summarization of data using different machine learning approaches. The application of machine learning techniques using a large data sets like EDHS provides invaluable and scientifically sounded information for policy makers^1,2^. Predictive analytics in health care can significantly reduce the burden of health professionals regarding patient diagnosis and treatment, which results in tremendous changes to the health system of developed as well as developing nations^2^. It can also be possible to improve the quality of evidence-based decision making. These new innovations in medical care have been expanding the accessibility of electronic data and opening new doors for decision support and productivity improvements.

In this study, we applied an advanced non-traditional data analysis model and nationally representative data in Ethiopia to fill the gaps of the traditional methods by detecting risk indicators and extracting applicable rules for users and decision-makers. Therefore, the aim of this study is to determine the predictors of anemia among under-five year’s age children in Ethiopia using a machine-learning approach.

## Methods

### Study design and setting

A cross-sectional study design was used among under-five children in Ethiopia using the 2016 Ethiopian demographic and health survey data.

### Sample size estimation and sampling techniques

The investigation was conducted from January 18 to June 27, 2016. The Ethiopian Demographic and Health Survey, which was finished in 2016, provided the information for this study. As a member of the worldwide Demographic and Health Surveys program, Ethiopia participated in the EDHS 2016 for the fourth time. The Central Statistical Agency (CSA) collected it at the Federal Ministry of Health's request (FMoH). Data were gathered using a standardized, previously verified questionnaire. During the EDHS interviews, interviewers utilized tablet computers to record replies. The tablets had Bluetooth technology installed to support distant electronic file transmission (transferring assignment sheets from team supervisors to interviewees and completed copies from interviewer to supervisor). A representative sample of over 18,008 households from 624 clusters spread throughout nine regions and two administrative cities were chosen by the EDHS.

Employing a two-stage cluster design, the 2016 EDHS sample was chosen, with the first stage using census enumeration areas (EAs) as sampling units. In a conventional two-level stratification, the population is first divided into regions, and then within each region, into urban and rural areas. The sample had 645 EAs (202 in urban areas and 443 in rural areas). Households made up the second round of samples in the sampling process. The 645 chosen enumeration areas underwent a comprehensive listing of homes employing equal probability systematic sampling with proportionate to EA.

**Figure 1:**
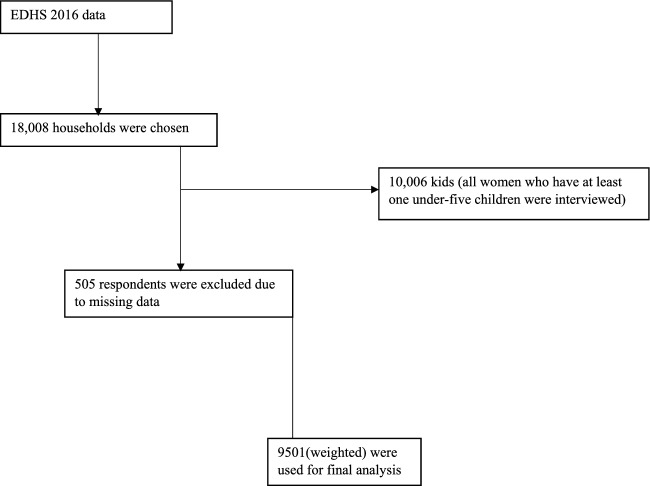
Sampling procedure for under-five children’s anemia prediction analysis.

Participation was open to all females between the ages of 15 and 49 who had at least one child in the five years prior to the survey. 10,006 kids under the age of five would make up the sample size for this study. A total of 9501 under-five children were included in this study after the missing values were deleted since the EDHS data contains numerous missing values for several variables (Fig. 1).

### Criteria for exclusion and inclusion

The under-five age children data was used in the 2016 EDHS data set. Respondents having insufficient data and missing values were excluded from this study.

### Study variables and measurements

The outcome variable was the presence or absence of anemia in a child under the age of five, and it was coded with a value of zero to denote the absence of anemia and a value of one to denote the presence of anemia. A hemoglobin or hematocrit value has been necessary for a kid to be identified as having anemia, which were measured based on mothers' complaints regarding the symptoms of these illnesses^24^.

### Data quality control

The data for this study was obtained from the 2016 EDHS secondary data. The data was extracted by following strictly required procedures as shown in the above figure one. Data cleaning was done carefully. EDHS 2016 quality of the data was determined primarily by the quality of the fieldwork, following appropriate steps that can enhance it considerably during data processing. Data entry and editing for inconsistencies were critical steps in this research to remove missing data. Each relevant and pertinent data pre-processing step have been performed to assure the quality of the data.

### Data processing

There are 25 features and 9501 instances in the extracted datasets. Data preprocessing techniques such as data cleaning, data transformation, handling, class imbalance, and feature selection methods were used because all of these features are not pertinent for creating a predictive model that can predict the anemia among children under the age of five in the case of Ethiopia. Mode imputation methods for categorical data were used to fill in the missing values. Manual removal of redundant data was done. Features with more categorical values, such as the source of drinking water, body mass index, wealth index, parents’ occupation, and the type of fuel they used were transformed into discrete values using binning discretization mechanisms. These features have multiple distinct values and need to be transformed for mining purposes. Then, the essential characteristics that were crucial for the following steps were chosen using the Boruta Algorithm feature selection approach (Fig. 3).

A total of 9501 instances with 14 attributes were taken into consideration for further analysis and prediction model construction after completing all necessary data preprocessing activities.

### Ethical approval and consent to participate

The researchers received the survey data approval letter from the USAID DHS program after registering with the link https://www.dhsprogram.com/data/dataset_admin/login_main.cfm and then the researchers of this study maintained the confidentiality and privacy of the data. We have obtained authorization letter from ICF to use this data and we attached the letter as an annex. The study does not require ethical approval because it was a secondary data analysis using the 2016 EDHS database. After receiving the data from the USAID–DHS program, the researchers in this study maintained the data’s anonymity. During the survey, informed consent was received from the study participants prior to the start of study. All methods were carried out in accordance with relevant guidelines and regulations.

## Results

### Descriptive results of the socio-demographic characteristics

From a total of 9501 study participants, 51.1% of them were males and nearly 81% of the respondents were living in a rural area. On the other hand, about 64% of the participants had no educational background at all. Nearly 37% of the respondents were the poorest regarding the family wealth index. Around 60 percent of the mothers had no work. Majority of the respondents, 21.1% of them had age ranging in 48–59-month age category (Table 1).

**Table 1:** Socio-demographic profiles of respondents in Ethiopia from January 18 to June 27, 2016 (N = 9501).

Variable	Frequency (N)	Percent (%)
Age of child		
< 6 month	1249	13.10
6–11 month	763	8.00
12–23 month	1824	19.20
24–35 month	1829	19.30
36–47 month	1824	19.20
48–59 month	2012	21.20
Sex of child		
Male	4852	51.10
Female	4649	48.90
Residence		
Urban	1814	19.10
Rural	7687	80.90
Mothers highest educational level		
No education	6089	64.10
Primary	2370	24.90
Secondary	669	7.00
Higher	373	3.90
Wealth index		
Poorest	3547	37.30
Poorer	1583	16.70
Middle	1309	13.80
Richer	1153	12.10
Richest	1909	20.10
Mothers’ occupation		
Not working	5668	59.70
Working	3833	40.30
Number of living children		
1–3	4758	50.10
4–6	3385	35.60
Above 6	1358	14.30

### Environmental characteristics of respondents

In this Ethiopian Demographic Health Survey, 5312 study respondents (55.9%) traveled a long distance to get health services. According to this survey, about 53% study participants did’t get improved drinking water source and nearly 87% of them did’t have improved toilet facility. Unfortunately, around 95% respondents did’t have health insurance according to this survey (Table 2).

**Table 2:** Environmental characteristics of respondents in Ethiopia from January 18 to June 27, 2016 (N = 9501).

Variable	Frequency (N)	Percent (%)
Type of cooking fuel		
Electricity	458	4.80
Charcoal	849	8.90
Wood	7613	80.10
**Others fuel	581	6.10
Type of toilet facility		
Improved	1170	12.30
Not improved	8331	87.70
Source of drinking water		
Not improved	5046	53.10
Improved	4455	46.90
Disposal of youngest child’s stools when not using toilet		
Not safe	8449	88.90
Safe	1052	11.10
Covered by health insurance		
No	8991	94.60
Yes	510	5.40
Distance to health facility		
Long	5312	55.90
Not long	4189	44.10
Child lives with whom		
Respondent	9332	98.20
Lives elsewhere	169	1.80

**Other Fuel: Kerosene, straw/shrubs/grass, agricultural crops.

### Nutritional and co-morbid characteristics among under-five children

Among the total participants, nearly 96% of kids breastfed and about 8532(89.8%) of the respondents had no history of diarrhea. However; 5613 (59%) of the kids and the majority of them, 8393(88.3%) did’t take Vitamin-A supplement and intestinal parasites’ drugs in the previous six months respectively. Regarding nutritional status, about 922 (9.7%) of the respondents were wasted and 3997 (42.1%) were stunted (Table 3).

**Table 3:** Nutritional and co-morbid characteristics of Anemia among under-five children in Ethiopia from January 18 to June 27, 2016 (N = 9501).

Variable	Frequency (N)	Percent (%)
Currently breastfeeding		
No	3238	34.10
Yes	6263	65.90
Stunting		
Normal	5504	57.90
Severe	3997	42.10
Drug for intestinal parasites		
No	8393	88.30
Yes	1108	11.70
Duration of breast feeding		
Ever breastfeed	9134	96.10
Never breastfeed	367	3.90
Wasting		
Normal	8579	90.30
Wasting	922	9.70
Vitamin A supplement		
No	5613	59.00
Yes	3888	40.90
Short, rapid breaths (ARI)		
No	9028	95.00
Yes	473	5.00
Had diarrhea		
No	8532	89.80
Yes	969	10.20

*ARI* antiretroviral infection.

**Figure 2:**
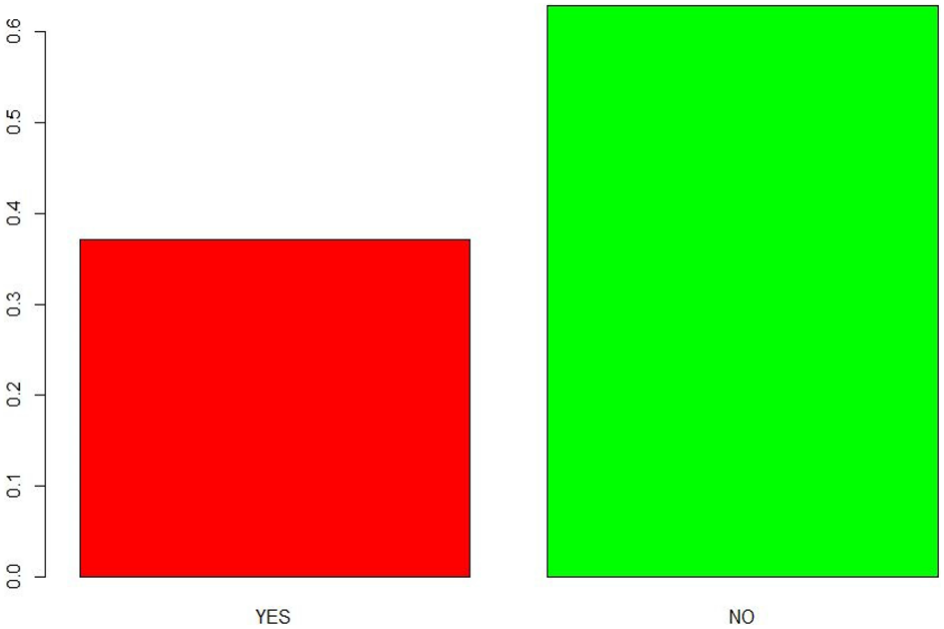
Prevalence of anemia among under-five age of children in Ethiopia.

The prevalence of anaemia among under-five children was nearly 40% in Ethiopia according to Ethiopian Demographic Health Survey Data (Fig. 2).

### Feature selection and determination of anemia’s predictors among under-five children

**Figure 3:**
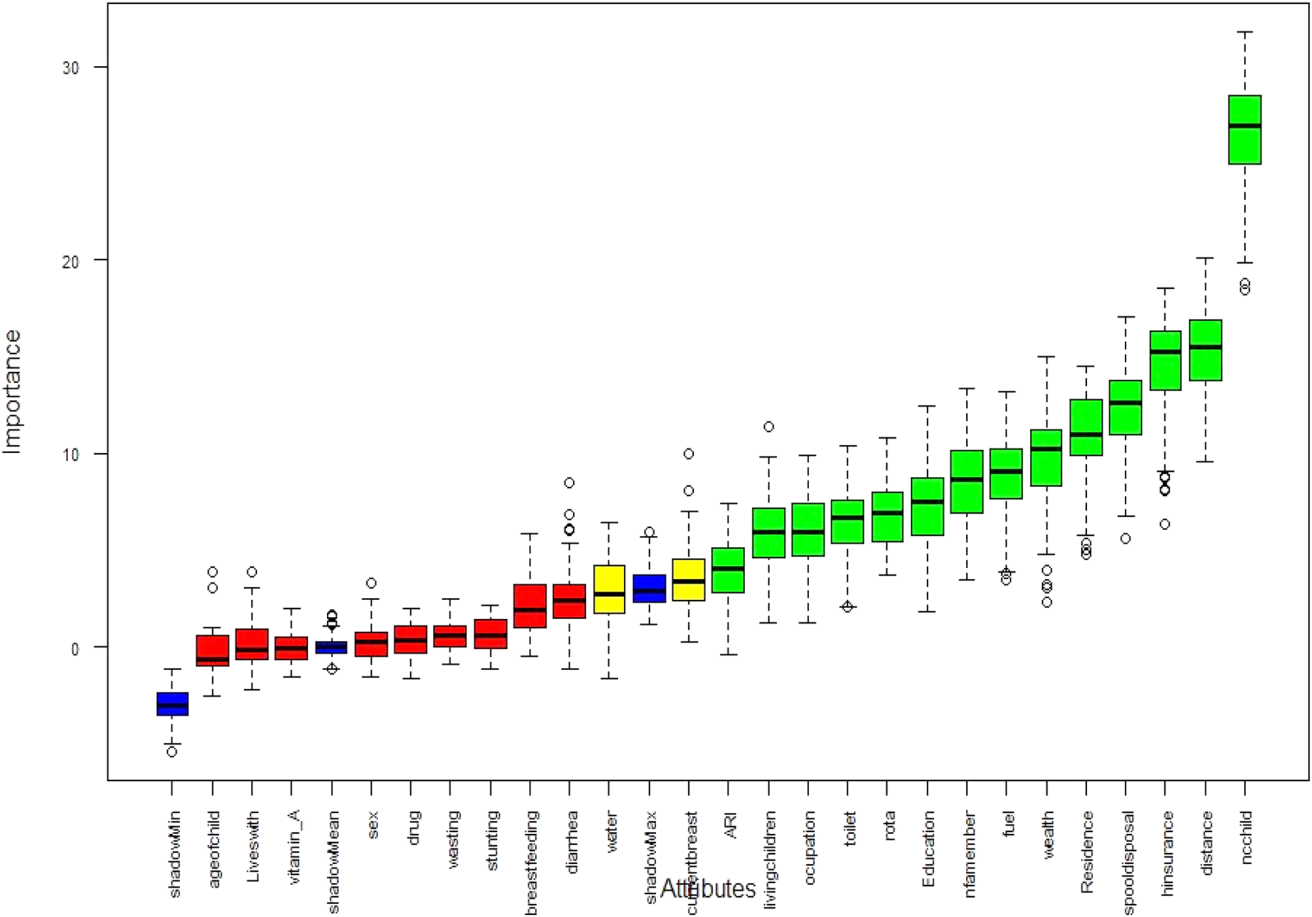
Feature selection using Boruta algorism for anemia variables’ feature selection.

The selection of features is an important phase in predictive modeling^25,26^. This method assumes utmost significance when a data set with a number of variables is provided for model construction. For this study, we used a Boruta feature selection algorism, a method that is common to apply when we are interested in understanding the mechanisms related to the variable of interest (Fig. 3). In the Fig. 3, variables in the boxplot sorted by increasing importance and colored in green are those which were classified as relevant and confirmed by the algorithm, and variables in red color are those which are irrelevant and rejected by the algorithm. The blue color boxplot indicated that shadow attributes which are created by the Boruta algorithm for benchmark or reference in variable importance comparing with category detection either the variables are relevant, tentative, or irrelevant.

Using the Boruta feature selection method, fourteen variables out of twenty-eight variables were selected as important features for model construction. Number of children, distance to health facility, health insurance coverage, youngest child’s stool disposal, residence, mothers’ wealth index, mothers’ educational status, occupation, type of toilet facility were some of the variables found to be significant for model building (Fig. 3). The variable description code for the Boruta Algorism has been shown in the following table (Table 4).

**Table 4:** Variable description codes for the above Boruta algorism figure.

Code of variable	Description
Ageofchild	Age of children
Livewith	Child lives with whom
Vitamin_A	Vitamin A Supplement
Sex	Sex of child
Drug	Drug for intestinal parasites
Wasting	Wasting
Stunting	Stunting
Nbreastfeeding	Duration of breast feeding
Diarrhea	Had diarrhea
Water	Source of drinking water
Curentbreast	currently breastfeeding
ARI	Antiretroviral infection
Livingchildren	Number of living children
Occupation	Mothers’ occupation
Toilet	Type of toilet facility
Rota	Received Rotavirus 1
Education	Mothers educational level
Nfamember	Number of family member
Fuel	Type of cooking fuel
Wealth	Mothers’ wealth index
Residence	Type of residence
Spooldisposal	Disposal of youngest child's stools when not using toilet
Hinsurance	Covered by health insurance
Distance	Distance to health facility
Ncchild	Number of living children

## Discussion

This study briefly described the prevalence of anemia and its predictors among under five children in Ethiopia using machine learning techniques. In this regard, the model showed that number of children, distance to health facility, health insurance coverage, youngest child’s stool disposal, residence, mothers’ wealth index, type of cooking fuel, number of family members, mothers’ educational status and receiving rotavirus vaccine were the top ten important predictors for anemia among under-five children.

The results of the ML model, in comparison, seem to be nearly in line with those of the conventional logistic regression analysis. It indicates that factors such as; distance to health facility, health insurance coverage, youngest child’s stool disposal, residence, mothers’ wealth index, type of toilet facility, number of family members, mothers’ educational status play a significant role in anemia level in children under the age of five in Ethiopia. In contrast to the usual logistic regression analysis, only receiving rotavirus vaccine appears to be relevant factor in ML models. This suggests that ML models could generate some "different variables" or now-unknown insights from the conventional regression models that could be essential in policy decision-making.

The findings of this study demonstrated a substantial relationship between anemia and health insurance coverage. This finding is in line with various studies conducted in Ghana^27–29^, where children who are not insured are at a higher risk of developing anemia compared to insured children. This is due the fact that health insurance serves as a strategy to acquire health services for health problems including anemia as easily as possible. Hence, uninsured households are at risk of financial shocks for health service costs, which leads to further progression of health problems including anemia and its treatment delay.

Surprisingly, there is a significant association between anemia and the type of cooking fuel. According to the findings of this study, children who are exposed to unclean cooking fuel and smoking are more likely to develop anemia than their counterparts are. This finding is supported by various studies conducted in Georgia State University^30^, India^31^ and survey of sub-Saharan countries^32^. This may be due to the reason that exposure to cooking fuel could lead to systemic inflammation mediated by inflammatory cytokines.

Similarly, the findings of this study revealed that household wealth status is a significant predictor of under-five child anemia. The occurrence of anemia among under-five children who are from rich household families is lower than children who are from poor families. This finding is in line with a study conducted in different locations of Ethiopia; Filtu town Somalia region^33^, southwest Ethiopia^34^,Wolaita^35^, Kombolcha^36^, South wollo^37^ and northeast^38^, University of Gondar^39^.It is also in agreement with studies conducted in Rwanda^40^, Bahir Dar University^41^,and Sudan^42^. The possible justification could be food scarcity, poor hygiene and sanitation, and poor childcare resulting in malnutrition including iron deficiency anemia.

According to this study, there is a significant relationship between anemia and receiving rotavirus vaccine among under-five children. Children who did not receive the rotavirus vaccine are more susceptible to develop anemia compared to those who receive the rotavirus vaccine. This is because children, which do not take the rotavirus vaccine, are at a higher risk of developing diarrhea that could result in the occurrence of anemia among under-five children^43^.Diarrhea could suppress the children’s immunity which exposes them to be vulnerable for other health problems resulting in nutritional deficiency including iron deficiency anemia.

Additionally, this study pointed out a significant association between the youngest child’s stool disposal and under-five children’s anemia. Children who disposed their stool improperly are high likely to develop anemia compared to their counterparts. This finding is in agreement with a study conducted in Tanzania^44^. This can be due to the exposure of children to helminths from improperly disposed stools. This could lead to a high chance of helminthic disease transmission like hook worm, which can lead to low food absorption, poor food appetite, gastro intestinal bleeding, and other complications, lastly resulting in anemia.

On the other hand, this study showed a substantial association between anemia and the number of family members and children, where children who are living in households having a large number of family sizes and children are at a higher risk of developing anemia compared to those children who are living with a small family size. This is in line with studies conducted in Southwest Ethiopia^34^ and University of Gondar^39^. This is because children living with a large number of children and family members might be in competition for foods and are easily susceptible to communicable diseases, which all entirely lead to nutritional deficiencies, particularly iron deficiency anemia.

Under-five children’s anemia was also shown to be predicted by the mother's educational level. This assertion is in line with past discoveries conducted in South Wollo^37^, Jimma University^39^, University of Gondar^39^, Uganda^21^, sub-Saharan countries^32^, Malawi^4^, and Rwanda^40^, Togo^45^ and Palestine^46^. This could be because education enables moms to manage their surroundings, including healthcare facilities, collaborate with medical experts more successfully, adhere to treatment recommendations, and maintain a clean environment. Furthermore, women with greater education have more influence over the health choices that their kids make.

Likewise, residence is significantly associated with under-five children’s anemia according to the findings of this study, where children who are living in rural resident are more at risk of developing anemia compared to their counterparts. This finding is supported by studies conducted in southwest Ethiopia^34^, Sudan^42^, Brazil^47,48^, Bolivia^49^. This is because low economic status, lack of iron-rich foods, lack of information about balanced dietary intake, and high number of illiterates could be linked with the occurrence of anemia among under-five children.

Moreover, the distance of health facility predicted the occurrence of anemia among under five children according to the findings of this study. Children who are living at a higher distance far from their health facility are at a higher risk of developing anemia. This is in line with a study conducted in University of Gondar^50^. This is due to the reason that children living at a longer distance far from their health facility might have inadequate health seeking behavior, which leads to delayed treatment for common childhood illness and lastly results in anemia following complicated health problems due to inaccessible health facilities.

Even though the importance of the variables is less compared with the other variables, mothers’ occupation, toilet facility, and antiretroviral infection were also figured as a predicting factor for anemia among under-five children. Finally, the ML findings appear to be mostly incomprehensible in contrast to the classical regression models since they lack regression coefficients and, consequently, a direction of impact. In practice, ML models often categorize or forecast particular factors depending on how significant a role they had in influencing the anemia level among children under the age of five in the current research. The direction of these crucial factors may be ascertained in this situation utilizing the available empirical literature from investigations employing conventional approaches. However, machine-learning methods are considered extremely helpful in predicting the determinants of population health and other phenomena resulting in policy decisions improvement^51,52^.

### Strengths and limitations of the study

Large and nationwide sample usage is the crucial strength of this study; this can enable this study to infer the results for the general population. The application of advanced machine learning prediction algorithm was another unique strength of the study. The limitation of this study was the restriction of researchers just to the variables found in the survey since the research is conducted using secondary data. Additionally, the analysis was only among under-five years old children.

## Conclusions

Machine-learning algorithm was applied to determine the predictors of anemia among under- five year’s age children in Ethiopia. To conduct this study, we have done twelve experiments. We have identified the determinant factors by conducting a feature importance analysis with the Boruta algorithm. Some of the most significant predictors were number of children, distance to health facility, health insurance coverage, youngest child’s stool disposal, residence, mothers’ wealth index, and type of cooking fuel. Machine learning model plays a paramount role for policy and intervention strategies related to anemia prevention and control among under-five children.

## Data Availability

The datasets used or analyzed during the current study are available from the corresponding author on reasonable request. The data was obtained with a link https://www.dhsprogram.com/data/dataset_admin/login_main.cfm.

## Abbreviations

CSA: Central statistical agency
EDHS: Ethiopian demographic and health survey
FMoH: Federal Ministry of Health
ML: Machine learning
SPSS: Statistical Package for Social Sciences
